# An Improved Procedure for *Agrobacterium*-Mediated Transformation of ‘Carrizo’ Citrange

**DOI:** 10.3390/plants11111457

**Published:** 2022-05-30

**Authors:** Yanjun Li, Dan Tang, Zongrang Liu, Jianjun Chen, Baoping Cheng, Rahul Kumar, Huseyin Yer, Yi Li

**Affiliations:** 1 Department of Plant Science, University of Connecticut, Storrs, CT 06269, USA; yanjun.li@uconn.edu (Y.L.); dan.tang@uconn.edu (D.T.); rahul.kumar@uconn.edu (R.K.); huseyin.yer@uconn.edu (H.Y.); 2 Horticulture and Landscape College, Hunan Agricultural University, Changsha 410128, China; 3 Appalachian Fruit Research Station, Agricultural Research Service, U.S. Department of Agriculture, Kearneysville, WV 25430, USA; zongrang.liu@usda.gov; 4 Mid-Florida Research and Education Center, Department of Environmental Horticulture, Institute of Food and Agricultural Sciences, University of Florida, Apopka, FL 32703, USA; jjchen@ufl.edu; 5 Plant Protection Research Institute, Guangdong Academy of Agricultural Sciences, Guangdong Provincial Key Laboratory of High Technology for Plant Protection, Guangzhou 510640, China; chengbaoping@gdass.cn

**Keywords:** treatments of *Agrobacterium* *tumefaciens* and explants, acetosyringone, 2-(*N*-morpholino) ethanesulfonic acid, paclobutrazol, lipoic acid

## Abstract

Although several protocols for genetic transformation of citrus have been published, it is highly desirable to further improve its efficiency. Here we report treatments of *Agrobacterium* cells and citrus explants prior to and during co-cultivation process to enhance transformation efficiency using a commercially used rootstock ‘Carrizo’ citrange [*Citrus sinensis* (L.) Osb. × *Poncirius trifoliata* (L.) Raf.] as a model plant. We found explants from light-grown seedlings exhibited higher transformation efficiency than those from etiolated seedlings. We pre-cultured *Agrobacterium* cells in a 1/10 MS, 0.5 g/L 2-(*N*-morpholino) ethanesulfonic acid (MES) and 100 µM acetosyringone liquid medium for 6 h at 25 °C before used to infect citrus explants. We incubated epicotyl segments in an MS liquid medium containing 13.2 µM 6-BA, 4.5 µM 2,4-D, 0.5 µM NAA for 3 h at 25 °C prior to *Agrobacterium* infection. In the co-cultivation medium, we added 30 µM paclobutrazol and 10 µM lipoic acid. Each of these treatments significantly increased the efficiencies of transformation up to 30.4% (treating *Agrobacterium* with acetosyringone), 31.8% (treating explants with cytokinin and auxin), 34.9% (paclobutrazol) and 38.6% (lipoic acid), respectively. When the three treatments were combined, we observed that the transformation efficiency was enhanced from 11.5% to 52.3%. The improvement of genetic transformation efficiency mediated by these three simple treatments may facilitate more efficient applications of transgenic and gene editing technologies for functional characterization of citrus genes and for genetic improvement of citrus cultivars.

## 1. Introduction

Citrus is one of the most important fruit crops in the world. Developing new citrus cultivars with improved yield and fruit quality has always been a top priority [1]. Compared with conventional breeding methods, genetic engineering technologies are more efficient alternatives to breed new citrus cultivars [2,3,4]. Additionally, the rapid development of bioinformatics tools and sequencing technologies have led to the identification of a large number of genes of interest from citrus accessions [5,6,7]. However, functions of many genes remain largely obscured. To validate gene functions, further improvement of citrus transformation efficiency is highly desirable. Although transgenic technologies have been successfully used for basic plant research and genetic improvement of agronomically important traits for decades; but concerns and issues associated with transgenic plant technologies have hampered their applications in citrus [8,9,10]. Recently developed gene-editing technologies provide a powerful and more acceptable tool for citrus improvement [11,12]. The use of gene-editing tools in citrus also needs genetic transformation system in most cases.

Different delivery methods have been developed for genetic transformation of citrus including polyethylene glycol (PEG)- [13], electroporation- [14] or particle bombardment- [15], and *Agrobacterium*-mediated [16] transformation. The bombardment method requires specific equipment, and PEG and electroporation methods need complex and difficult procedures for plant regeneration from protoplasts. Among these methods, the *Agrobacterium*-mediated method is easiest and most convenient [17].

Because ‘Carrizo’ citrange is an economically important and widely used rootstock in the citrus industry worldwide, it has been used as a model plant for citrus transformation. Efforts have been made to improve *Agrobacterium*-mediated citrus transformation efficiency. For instance, Yu et al. [18] found that using longitudinal cutting explants derived from 3-week-old ‘Carrizo’ citrange etiolated seedlings increased the citrus transformation efficiency. Attempts have been made by Dutt and Grosser to improve the citrus transformation method through optimizing acetosyringone concentrations and co-cultivation time, resulting in a relatively high transformation efficiency in ‘Carrizo’ citrange [19]. Transformation efficiencies of ‘Carrizo’ citrange were improved by the use of isopentenyl transferase gene (*IPT*) and knotted1 gene (*KN1*) [20]. In 2015, Orbovic and Grosser [21] reported 7.8% transformation efficiency for ‘Carrizo’ citrange, which appears to be an average transformation efficacy for ‘Carrizo’ citrange observed by many investigators [19,20,21,22,23].

In this study, we also used ‘Carrizo’ citrange as model plant to improve citrus transformation. Here we report that using light-grown seedlings as epicotyl explant sourceplus some simple treatments for *Agrobacterium* and epicotyl explants prior to and during infection: (1) culturing *Agrobacterium* with acetosyringone, (2) culturing explants in cytokinin and auxin enriched medium, and (3) addition of paclobutrazol and lipoic acid in the co-cultivation medium. We have observed a drastic improvement of transformation efficiency of ‘Carrizo’ citrange. The combination of the three treatments synergistically enhanced transformation efficiency by 5 folds.

## 2. Results

### 2.1. Explants from Light-Grown Seedlings Exhibited Higher Transformation Efficiency than Those from Etiolated Seedlings

**Figure 1:**
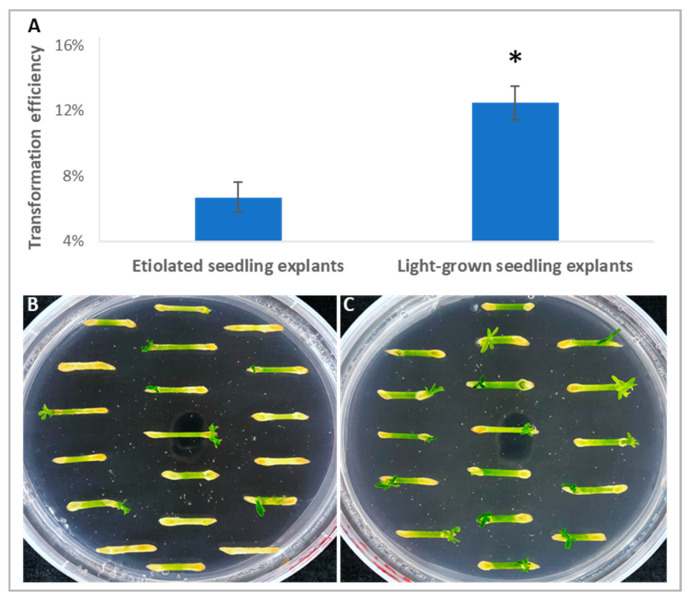
Light-grown explants of ‘Carrizo’ citrange had higher transformation efficiency compared with etiolated explants. (**A**) Higher transformation efficiency was observed with light-grown epicotyl explants compared with etiolated epicotyl explants. Asterisk represents significant difference between etiolated seedling explants and light-grown seedling explants (*p* < 0.05, ANOVA). (**B**,**C**) Shoots regeneration from epicotyl explants derived from etiolated seedlings (**B**), and from those of light-grown seedlings (**C**) at day 30th post-*Agrobacterium*-infection (dpi).

Epicotyl explants derived from both etiolated and light-grown seedlings of ‘Carrizo’ citrange were used to determine their transformation efficiencies. We harvested adventitious shoots produced at day 30th post-*Agrobacterium*-infection. GUS histochemical staining was used to conveniently determine which regenerated shoots were transgenic. Additional confirmation of transgenic shoots was described in a later section. Based on the GUS activity assay, we observed that transformation efficiency for explants from light-grown seedlings was 12.5%, while only 6.7% for explants from etiolated seedlings (Figure 1A–C). We also noticed that most shoots regenerated from the etiolated explants that turned green during the shoot regeneration process. However, some etiolated explants never turned green, and these explants did not produce shoots (Figure 1B), which may contribute to the lower transformation efficiency observed. Accordingly, all subsequent experiments were performed with light-grown seedlings.

### 2.2. Treatments of Agrobacterium Cells and ‘Carrizo’ Citrange Explants Prior to and during Co-Cultivation on Citrus Transformation Efficiency

**Figure 2:**
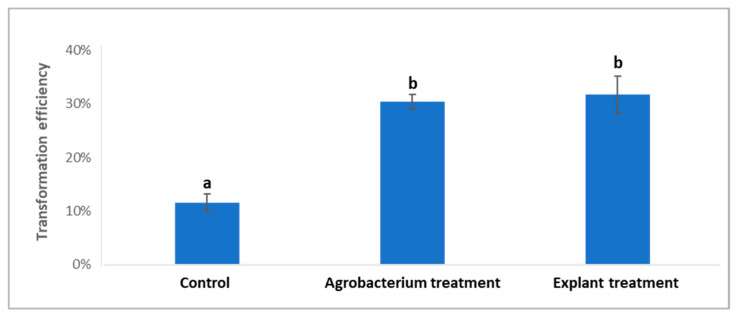
Treatments of *Agrobacterium* and epicotyl explants of ‘Carrizo’ citrange prior to co-cultivation increased citrus transformation efficiencies. The different letters represent significant difference among different treatments (*p* < 0.05, ANOVA, Tukey/LSD).

T-DNA transfer into plant nucleus and integration into plant genome is dependent on *vir* genes expression [24], which should affect transformation efficiency of citrus. To test whether a *vir* induction treatment could improve the transformation efficiency, we added an additional step by treating *Agrobacterium* cells with a plant tissue culture medium (1/10 MS, 0.5 g/L MES (a buffer)) plus 100 µM acetosyringone for 6 h before used for infection (the *Agrobacterium* treatment). Acetosyringone is an inducer for *vir* gene expression [25]. Figure 2 shows that *Agrobacterium* cells treated with the *vir* induction medium significantly increased the transformation efficiency from 10.1% to 30.4%.

We also tested if the treatment of light-grown seedling explants with cytokinin and auxins before *Agrobacterium* infection could improve transformation efficiency. Explants were incubated in a solution containing 13.2 µM 6-BA, 4.5 µM 2,4-D, 0.5 µM NAA [19] for 3 h at 25 °C before co-cultivation (the explant treatment). Figure 2 shows that cytokinin and auxin-treated explants enhanced transformation efficiency to 31.8% compared to 10.1% in non-treated explants.

**Figure 3:**
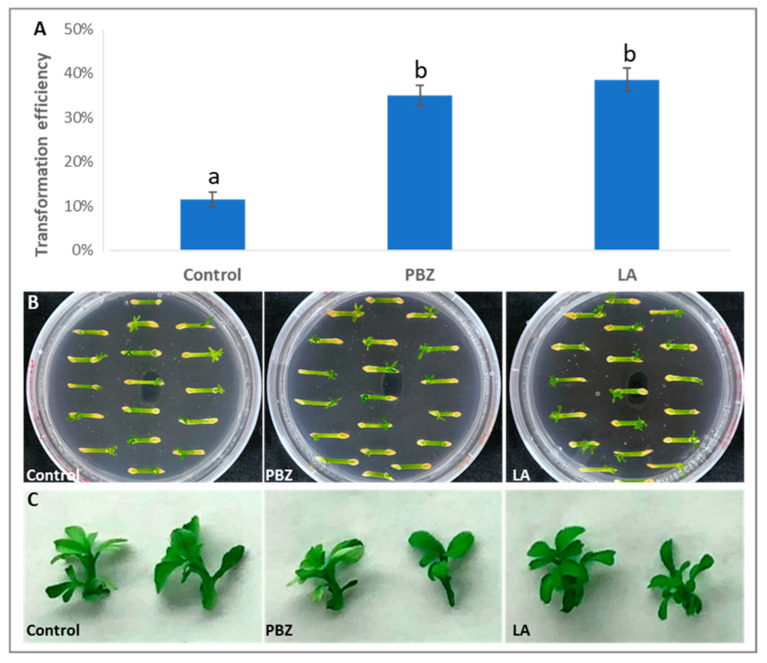
Chemical treatments during co-cultivation increased transformation efficiencies of ‘Carrizo’ citrange. (**A**) Paclobutrazol (PBZ) and lipoic acid (LA) treatments increased transformation efficiencies, respectively. Different letters represent significant difference among different treatments (*p* < 0.05, ANOVA, Tukey/LSD). (**B**) Shoot regeneration under kanamycin selection with different chemical treatments 30th post-*Agrobacterium*-infection. (**C**) Shoots produced from paclobutrazol and lipoic acid treatments 60th post-*Agrobacterium*-infection displayed no obvious differences in morphology.

Given that both paclobutrazol and lipoic acid were able to enhance transformation efficiency in various plant species [26,27,28], we added 30 µΜ paclobutrazol or 10 µΜ lipoic acid into co-cultivation media, respectively. The paclobutrazol treatment showed increased transformation efficiency from 11.8% to 34.9% (Figure 3A). Similarly, lipoic acid treatment also produced a similar effect (Figure 3A). These two treatments did not alter development or morphology of shoots (Figure 3B,C).

### 2.3. Incorporation of the Three Simple Treatments into Transformation Procedure and Analysis of Their Effects on Transformation Efficiency in ‘Carrizo’ Citrange

To examine if combining the three simple treatments could synergistically increase transformation efficiency, we tested two combinations. The first is the treatment of *Agrobacterium* with acetosyringone and the light-grown seedling explants with cytokinin and auxins prior to the *Agrobacterium* infection. The second is the same as the first but with the addition of paclobutrazol and lipoic acid into the *Agrobacterium* co-cultivation medium (Table 1). The first combination led to 258% transformation efficiency (Table 1), suggesting no synergistic effect between the *Agrobacterium* and explant treatments prior to the *Agrobacterium* infection (Figure 2). However, in the second combination, the transformation efficiency was elevated to 452.34%, indicating the addition of paclobutrazol and lipoic acid synergistically enhanced transformation efficiency.

### 2.4. Confirmation of Transgenic Shoots by PCR Amplification

**Figure 4:**
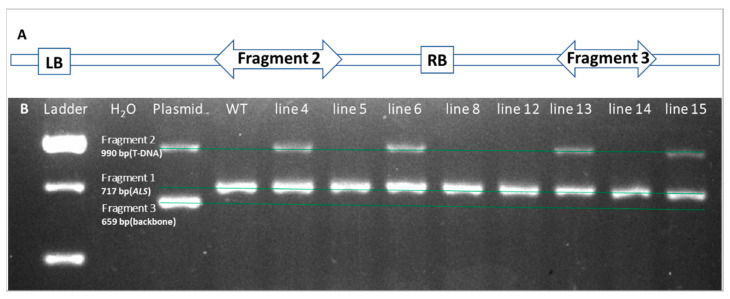
PCR confirmation of transgenic shoots of ‘Carrizo’ citrange based on the histochemical staining of GUS activity. (**A**) Two sets of PCR primers to amplify a T-DNA region (Fragment 2) and a backbone region (Fragment 3) used to confirm GUS positive shoots are transgenic. (**B**) Gel electrophoresis analysis of PCR products to confirm stable incorporation of transgenes into the citrus genome. The presence of both Fragment 1 and 2 and the absence of Fragment 3 were indicative of transgenic (Shoot lines 4, 6, 13, and 15). The presence of only fragment 1 was indicative of non-transgenic (Shoot lines 5, line 8, 12 and 14). Lane 1: Ladder. Lane 2: blank control (H_2_O as PCR template). Lane 3: positive control (plasmid as PCR template). Lane 4: negative control (WT citrus genomic DNA as template).

To further verify if the GUS positive shoots were transgenic, we randomly chose 42 GUS positive shoots, 7 GUS negative (presumably escaped shoots from kanamycin selection) shoots and WT shoots for PCR analysis. The representative results are shown in Figure 4B, which were based on the analysis of three sets of PCR primers. The first set of primers was used to amplify a fragment of the citrus *ALS* gene, which served as an indication that the PCR reaction works well. We named the *ALS* gene fragment as Fragment 1. The second set of primers was used to amplify a fragment within the T-DNA region of the Ti-plasmid, named as Fragment 2 (Figure 4A). The third set of primers was used to amplify a fragment in the backbone region of the Ti-plasmid, named as Fragment 3 (Figure 4A). Figure 4B shows that the PCR reactions worked well because Fragment 1 was always presented in all shoot samples. The presence of Fragment 2 (a T-DNA region) but the absence of Fragment 3 (a backbone region) confirmed that T-DNA was integrated into the citrus genome of these shoots. On the other hand, the presence of both Fragments 2 and 3 indicated that there were *Agrobacterium*/Ti-plasmid contaminations. In that case, the presence of Fragment 2 might not necessarily indicate that a particular shoot was transgenic. As shown in Figure 4B, no Fragment 3 was detected in all shoot samples, indicating no *Agrobacterium*/Ti-plasmid contaminations in the shoot samples. Figure 4B shows shoot tissue samples 5, 8, 12, and 14 had no Fragment 2 or 3 detected, which was consistent with the GUS negative results and therefore confirmed these shoots were non-transgenic. On the other hand, shoot samples 4, 6, 13 and 15 had Fragment 2 detected, which is consistent with the GUS positive results and therefore confirmed these shoots were transgenic. We observed our PCR data were 100% consistent with the results of histochemical staining assay of GUS activity in these shoot samples (Table 2), demonstrating GUS activity assay is reliable to identify transgenic shoots in our study.

## 3. Discussion

In this study, we improved the transformation efficiency of ‘Carrizo’ citrange with three simple treatments using light-grown seedlings as explant source. Our experiments demonstrate that using explants from light-grown seedlings for transformation leads to a higher transformation efficiency. Among the three treatments, one is to treat *Agrobacterium* cells in a diluted MS liquid medium with acetosyringone for 6 h right before they are used to infect citrus explants. The second treatment is to incubate the epicotyl explants in a liquid medium with cytokinin and auxins for 3 h immediately before they are infected with *Agrobacterium* cells. The third is to include paclobutrazol and lipoic acid in the co-cultivation medium. A combination of the three treatments led to the increase in transformation efficiency from 11.5% to 52.3%.

Earlier studies used both etiolated seedlings and light-grown seedlings for citrus transformation [18,19,20,21,22,23]. Our study shows higher transformation efficiency can be achieved using explants derived from light-grown seedlings, consistent with the study in kumquat that a relatively higher transformation efficiency was obtained when light-grown seedlings were used as explant source [29]. It is known that light reduces auxin levels in plant tissues [30,31]. We previously demonstrated that reduction in endogenous auxin levels in citrus explants can enhance shoot regeneration [32]. Thus, the higher transformation efficiency with explants from light-grown seedlings could be partially due to light-mediated reduction in endogenous auxin level and therefore leads to enhancement of shoot regeneration from transformed cells as we have previously reported [32].

*Agrobacterium vir* genes play a significant role in transfer of T-DNA into plant nucleus and integration of T-DNA into plant genome [24]. Acetosyringone has been used to induce *vir* genes expression and enhance plant transformation efficiency [33,34,35,36]. Hence, an additional step to treat *Agrobacterium* using acetosyringone to induce *vir* genes expression before the explant infection appears to be helpful to increase the transformation efficiency. Our treatment is to incubate *Agrobacterium* cells in a 1/10 MS (pH 5.6) with 100 µM acetosyringone at 25 °C for 6 h immediately before used for infecting citrus explants.

The treatment of explants with cytokinin and auxins before *Agrobacterium* infection has been reported to increase transformation efficiency in a few plant species [37,38]. For example, benzyladenine (BA), indole-3-acetic acid (IAA) and naphthaleneacetic acid (NAA) treatments of explants have been shown to promote cell division and growth in Troyer citrange [38], of which cytokinin is most effective to stimulate cell competence in shoot regeneration [32]. In this study, the treatment of explants in a medium containing cytokinin and auxin for 3 h prior to *Agrobacterium* infection has led to an increase competence of cell division and growth and therefore promoted the transformation efficiency (Figure 2).

Lipoic acid is a potent plant transformation enhancer that increased transformation efficiency in soybean, tomatoes, wheat, and cotton [26,27]. Paclobutrazol has also been shown to enhance transformation efficiency in *Petunia* hybrid [28]. We showed that both chemicals increased citrus transformation efficiencies (Figure 3). However, how these chemicals work in promoting transformation efficiency remains unclear. Paclobutrazol has been implicated multi-stress ameliorant [39,40], which may weaken plant defense against *Agrobacterium*. On the other hand, paclobutrazol was reported as a plant growth regulator [41] and was shown to increase callus formation and embryogenesis [42,43]. Thus, paclobutrazol treatment may have dual roles. The promotional effect of lipoic acid on citrus transformation efficiency may be ascribed to its protective role [44]. Previous studies demonstrated that *Agrobacterium* infection can trigger tissue browning and cell death, resulting in reduction of transformation efficiency [45]. The use of lipoic acid can reduce the browning and necrosis on *Agrobacterium* infected explants, which significantly improved transformation efficiency [26,27].

**Figure 5:**
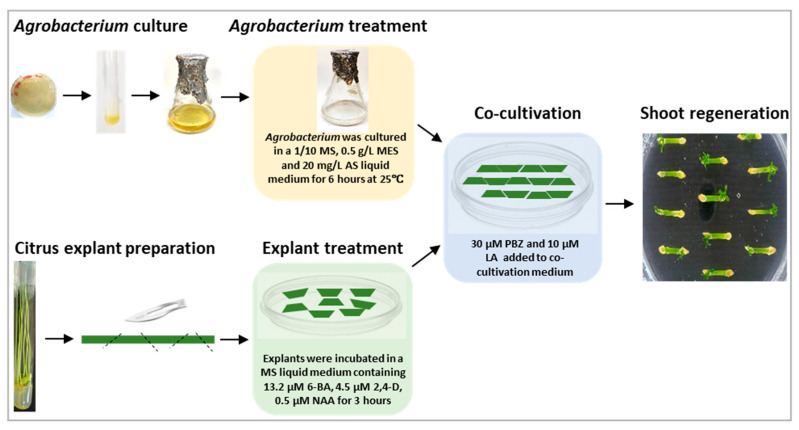
An outline of an improved procedure for *Agrobacterium*-mediated citrus transformation. Three simple treatments described in colored boxes were added to a conventional procedure for *Agrobacterium*-mediated citrus transformation, and these treatments significantly enhanced genetic transformation efficiency for ‘Carrizo’ citrange. AS: acetosyringone, MES: 2-(*N*-morpholino) ethanesulfonic acid, PBZ: paclobutrazol and LA: lipoic acid.

The combination of the three treatments synergistically enhanced transformation efficiency by 4 to 5 folds compared to the most widely used protocol for citrus transformation [21]. Based on our results, we conclude that the three treatments, that are (1) culturing *Agrobacterium* with acetosyringone, (2) culturing light-grown citrus epicotyl segments in cytokinin and auxin enriched medium, and (3) addition of paclobutrazol and lipoic acid in the co-cultivation medium, can significantly increase the transformation efficiency of ‘Carrizo’ citrange. The improved transformation is schematically represented in Figure 5. The *Agrobacterium* and explant treatments, as well as the use of paclobutrazol and lipoic acid in co-cultivation medium, as reported here, may also be useful for improving genetic transformation efficiencies in those plants that are recalcitrant to regeneration and infection. This improved transformation procedure may facilitate a more efficient characterization of citrus gene functions and the development of agronomically important traits in citrus using gene-editing technologies.

## 4. Materials and Methods

### 4.1. Plant Materials Preparation

‘Carrizo’ citrange [Citrus. *Sinensis* (L.) Osbeck × *Poncirus trifoliata* (L.) Raf.] seeds were purchased from Tree Source Citrus Nursery (504 N Kaweah Ave, Exeter, CA 93221, U.S.). Outer seed coats were removed manually, and seeds were surface sterilized with 75% alcohol for 1 min followed by 1% sodium hypochlorite for 20 min. After surface disinfection, all seeds were rinsed four times with sterile distilled water. Internal seed coats were removed under sterile conditions. Then, seeds were in vitro cultured on an MS medium with 30 g/L sucrose and 7 g/L agar at pH 5.7. Seeds were kept at 28 °C in the dark for 3 weeks before being transferred to light conditions with a photosynthetic photon flux density (PPFD) of 60 μmol/m^2^/s for another week unless stated otherwise. The photoperiod was 16 h.

To test the effects of dark- and light-grown seedlings on stable transformation efficiency, we cultured citrus seeds under different light/dark conditions. For etiolated seedlings, seeds were germinated in dark for 4 weeks before *Agrobacterium* transformation experiments. For light-grown seedlings, seeds were germinated in dark for 3 weeks and then transferred to light condition (60 μmol/m^2^/s) with a 16-h photoperiod for an additional week to let etiolated seedlings turn green.

To test the effect of hormone treatment of ‘Carrizo’ citrange explants on stable transformation efficiency, 1-cm-length epicotyl segments were incubated in an MS [46] liquid medium containing 13.2 µM 6-BA, 4.5 µM 2,4-D and 0.5 µM NAA for 3 h at 25 °C prior to *Agrobacterium* infection.

### 4.2. Agrobacterium Preparation

*Agrobacterium* tumefaciens strain EHA 105 harbors the binary vector pBin19 with the 35S-*nptII::uidA*. The *nptII* gene (kanamycin resistance gene) served as a marker gene for transgenic plants selection. The *uidA* (β-glucuronidase gene) served as a reporter gene for transgenic plants screening. The *Agrobacterium* stock was streaked on a solid LB [47] medium containing 100 mg/L kanamycin and 50 mg/L rifampicin and cultured for two days at 28 °C. Single colonies were transferred into a 5 mL liquid LB medium with 100 mg/L kanamycin and 50 mg/L rifampicin and cultured under 200 rpm, at 28 °C for 24 h. After that, 2 mL cultivated bacteria solution was transferred into a 50 mL liquid LB medium containing the same antibiotics and cultured with the same conditions to an OD_600_ of 0.6. *Agrobacterium* cells were then centrifuged at 5000 rpm for 15 min and resuspended in a liquid co-cultivation medium consisting of MS, 30 g/L sucrose and 100 µM acetosyringone, which is ready for infection.

To test acetosyringone treatment of *Agrobacterium* cells on stable transformation efficiency, the harvested *Agrobacterium* cells were resuspended in a medium containing 1/10 MS, 0.5 g/L MES, and 100 µM acetosyringone and cultured at 25 °C for 6 h before co-cultivation.

To test if combining the *Agrobacterium* treatment with acetosyringone and the explant treatment with cytokinin and auxins prior to the *Agrobacterium* infection could synergistically increase transformation efficiency, we did combination with these two treatments.

### 4.3. Agrobacterium-Mediated Citrus Transformation and Citrus Shoot Regeneration

On the day of *Agrobacterium* infection, citrus epicotyl was cut into 1 cm segments in a sterile condition and incubated in the *Agrobacterium* cells suspension basically as described previously [21]. After being blotted dry on sterilized filter paper, explants were placed horizontally in Petri dishes containing solid co-cultivation medium consisting of MS, 13.2 µM 6-BA, 30 g/L sucrose and 100 µM acetosyringone unless stated otherwise and incubated at 25 °C in the dark. After three days co-cultivation, explants were transferred to a shoot regeneration medium containing MS, 13.2 µM 6-BA, 30 g/L sucrose and 8 g/L agar. All shoot regeneration media were supplemented with 100 mg/L kanamycin and 150 mg/L timentin. The explant tissues were cultured under a light condition (60 µmol/m^2^/s) at a 16-h photoperiod (26 ± 2 °C) and were transferred onto fresh shoot regeneration media every 3 weeks.

To test the effects of paclobutrazol and lipoic acid treatments on stable transformation efficiencies, 30 µM paclobutrazol and 10 µM lipoic acid were investigated by directly adding to the co-cultivation media, respectively.

**Figure 6:**
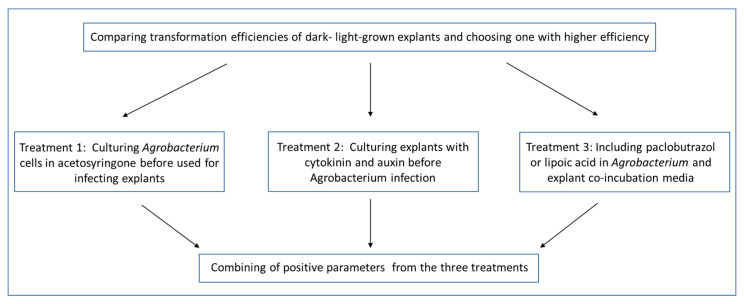
A flowchart of testing different parameters to improve transformation efficiency of ‘Carrizo’ citrange.

To examine if combination of these simple treatments could synergistically increase transformation efficiency, we also tested the effects of their combination on transformation efficiency. A flow chart of our experimental design is shown in Figure 6.

### 4.4. GUS Histochemical Assays

GUS staining solution consisting of 100 mM potassium phosphate buffer, 10 mM Na_2_EDTA, 0.5 mM K_3_Fe(CN)_6_, 0.5 mM K_4_Fe(CN)_6_, 0.1% Triton X-100, 1 g/L X-gluc(5-bromo-4-chloro-3-indolyl-b-D-glucuronic acid) was prepared in advance and stored at 4 °C for a few weeks. For histochemical assays of GUS activity, small leaf slices of regenerated shoots longer than 5 mm were cut and soaked in the GUS staining solution and incubated at 37 °C for 16 h. After that, plant tissues were destained in ethanol to gradually remove chlorophylls and other pigments prior to being visually inspected.

### 4.5. Molecular Confirmation of Transgenic Shoots

Three sets of primers were designed for verification of stable transformation events. The first set of primer pairs (*GUS*_F: 5′-CCGGGTGAAGGTTATCTCTATG and *GUS*_R: 5′-GCGAGTGAAGATCCCTTTCT) was used to specifically amplify a 990-bp GUS coding fragment, which is located within the T-DNA region of the binary vector. The second set of primer pairs (Out_F: 5′-CCTCGCAGAGATCCGAATTATC and Out_R: 5′-TAGGTAGCCCGATACGATTGA) was used to specifically amplify a 659-bp fragment located outside the T-DNA region, but within the backbone region of the binary vector. Inclusion of this primer pairs is to discriminate non-integrated binary vector from the T-DNA integrated event on genome. The third set of primer pairs (*ALS*_F: 5′-ATACCGAAAGGTTGGGCAGG; and *ALS*_R: 5′-TCACCACGATGCCATGTTCA) was used to amplify a 717-bp DNA fragment from citrus genome.

Genomic DNA samples were isolated from both GUS-positive and GUS-negative shoots (GUS-silenced transgenic lines or escaped WT shoots) under kanamycin selection. PCR reactions were performed at a 20 μL volume containing 1× PCR buffer (Takara, Japan), 1.5 mM MgCl_2_, 0.25 mM dNTPs, 0.2 μL e2TAK DNA polymerase (Takara, Japan), 0.25 mM of each primer and 500 ng genomic DNA as template, with an initial denaturation at 98 °C for 5 min, followed by 35 cycles of 98 °C for 10 s, 65 °C for 5 s, and 72 °C extensions for 1 min plus a final extension at 72 °C for 10 min. PCR products were separated by electrophoresis on 2% (*w*/*v*) agarose gels.

### 4.6. Data Analysis

All experiments were repeated three times with above 200 explants per replication in each treatment. Transformation efficiency (%) was calculated as following: the total number of transgenic shoots/total number of explants × 100% in each treatment.

Statistical analysis was performed with SPSS software. Each result was presented as the mean ± standard deviation (SD) of at least three replicated measurements. We conducted the significant difference using ANOVA. The significant difference (*p* < 0.05) between two treatments was compared using ANOVA (one-way analysis of variance). For multiple comparison analysis, Tukey’s test (as post hoc test) was applied followed one-way ANOVA for significant difference (*p* < 0.05) among different treatments.

For Figure 1A, significance was measured with one-way ANOVA analysis. F = 34.9. The *p*-value for comparing etiolated seedling with light-grown seedling is 0.0041 (*p* < 0.05).

For Figure 2, significance was measured with one-way ANOVA with Tukey’s multiple comparisons test; F = 120.7. The *p*-value for comparing control with *Agrobacterium* treatment is 0.00007 (*p* < 0.0001). The *p*-value for comparing control with explant treatment is 0.00029 (*p* < 0.05). The *p*-value for comparing *Agrobacterium* treatment with explant treatment is 0.4233 (*p* > 0.05).

For Figure 3A, significance was measured with one-way ANOVA with Tukey’s multiple comparisons test; F = 185.9. The *p*-value for comparing control with PBZ treatment is 0.00003 (*p* < 0.0001). The *p*-value for comparing control with LA treatment is 0.00007 (*p* < 0.0001). The *p*-value for comparing PBZ treatment with LA treatment is 0.1030 (*p* > 0.05).

For Table 1, significance was measured with one-way ANOVA with Tukey’s multiple comparisons test; F = 119.7. The *p*-value for comparing control with combination treatment 1 (*Agrobacterium* and explant combination treatment) is 0.00257 (*p* < 0.05). The *p*-value for comparing control with combination treatment 2 (*Agrobacterium* and explant treatment with PBZ and LA treatment) is 0.0000012 (*p* < 0.0001). The *p*-value for comparing combination treatment 1 with combination treatment 2 is 0.00354 (*p* < 0.05). We also applied LSD (as prior test) to compare ANOVA results for significant differences (*p* < 0.05) among different treatments. Both methods gave the same results about significance among different treatments.

## Figures and Tables

**Table 1:** Transformation Efficiencies of ‘Carrizo’ Citrange with Treatments Combined.

	Transformation Efficiency (%) ^1^	Compared with Control
Control	11.53 ± 1.68 ^a^	100%
*Agrobacterium* + explant treatment	32.80 ± 5.45 ^b^	258%
*Agrobacterium* + explant treatment + PBZ + LA ^2^	52.34 ± 1.10 ^c^	452%

^1^ Transformation efficiency was calculated based on the number of transgenic shoots recovered and the number of explants used (# transgenic plants per explant × 100%). Values followed by the different letters are significantly difference among different treatments (*p* < 0.05, ANOVA, Tukey/LSD). ^2^ PBZ: paclobutrazol and LA: lipoic acid.

**Table 2:** Transgenic Plants Confirmed by PCR ^1^.

PCR Experiment	No. of Tested Shoots		No. of PCR Confirmed Transgenic Shoots		Confirmation Rate (%)	
	GUS Positive	GUS Negative	From GUS Positive	From GUS Negative	From GUS Positive	From GUS Negative
1	15	3	15	0	100	0
2	14	2	14	0	100	0
3	13	2	13	0	100	0

^1^ We randomly chose 10–15 GUS positive shoots from each experiment for the PCR sassy.

## Data Availability

All data included in the main text.
